# No significant effect of mortality salience on unconscious ethnic bias among the Japanese

**DOI:** 10.1186/s13104-023-06360-9

**Published:** 2023-05-26

**Authors:** Kai Otsubo, Hiroyuki Yamaguchi

**Affiliations:** 1 Graduate School of Human-Environment Studies, Kyushu University, Fukuoka, Japan; 2 Faculty of Human-Environment Studies, Kyushu University, Fukuoka, Japan

**Keywords:** Terror management theory, Mortality salience, Worldview defense, In-group bias, Implicit Association Test

## Abstract

**Objective:**

Terror management theory posits that when mortality is salient, individuals attempt to defend their cultural worldviews. Although numerous studies have confirmed this hypothesis, some recent studies have suggested that East Asians do not engage in worldview defense. We conducted a pre-registered experiment with 895 Japanese adults to investigate whether they exhibited worldview defense at an unconscious level. Participants performed the Implicit Association Test using Japanese and Korean surnames as stimuli after being primed with thoughts about mortality.

**Results:**

The results revealed that mortality salience had no influence on implicit ethnic bias. These findings support the notion that East Asians do not engage in worldview defense, in accord with recent criticism of the validity of terror management theory. We discuss the limitations and implications of our findings.

**Supplementary Information:**

The online version contains supplementary material available at 10.1186/s13104-023-06360-9.

## Introduction

Terror management theory (TMT) [1] posits that mortality salience (MS) prompts individuals to defend their cultural worldviews (i.e., worldview defense). Although numerous experimental studies have confirmed this hypothesis [2, 3], some recent studies have questioned its validity [4, 5]. Most research regarding TMT has been conducted in Western countries, and an investigation of the validity of TMT predictions in East Asia is needed. Yen and Cheng [6] reported that Taiwanese participants did not exhibit worldview defense and found that the effect size of worldview defense in previous studies conducted in East Asian countries did not significantly differ from zero. In Japan, Toya and Nakashima [7] failed to replicate the results of Heine et al. [8], who reported a worldview defense effect in a Japanese sample.

In Yen and Cheng [6] and Toya and Nakashima [7], an evaluation of those who wrote anti-worldview essays was adopted as an indicator of worldview defense. It is possible that this methodological procedure led to the absence of a measurable worldview defense effect. Cultural psychologists have discovered that while independent self-construal is predominant in Western societies, interdependent self-construal emphasizing harmonious relationships with others is common in Eastern societies [9]. Previous research pointed out that this difference could influence a worldview defense effect among the Japanese [10]. TMT posits that MS motivates individuals to adapt to the criteria of their worldviews. Accordingly, it can be assumed that when mortality is salient, people with interdependent self-construal will exhibit tolerance of others, even when the target has insulted their worldviews. Indeed, Yanagisawa et al. [10] found that individuals with highly interdependent self-construal tended to evaluate those who wrote anti-Japanese essays more positively after MS priming. Importantly, their neurophysiological analysis indicated that individuals with highly interdependent self-construal attempted to exercise self-control and behave in a socially acceptable manner when faced with MS. The worldview defense effect in previous studies may have been concealed by such conscious self-control efforts. Thus, to verify the worldview defense effect among East Asians, it is necessary to conduct an experiment with measures that are not influenced by conscious self-control.

In the current study, we examined the MS effect on unconscious ethnic bias among Japanese individuals using the Implicit Association Test (IAT) [11], which was developed to measure implicit attitudes. We hypothesized that MS would intensify unconscious ethnic bias among the Japanese (H1). Using the IAT, Bradley et al. [12] demonstrated that MS intensified implicit racial bias against Black Americans among White American participants. If the assumptions of TMT hold irrespective of regionality, this result should be replicable. Some previous studies conducted in Japan support this hypothesis. Nodera et al. [13] showed that MS strengthened implicit gender stereotypes among male Japanese participants. In addition, Watanabe and Karasawa [14] found that MS provoked conceptual overlap between the self and the in-group in a Japanese sample. These studies suggest a worldview defense effect on unconscious ethnic bias, even in Japanese individuals. Additionally, we investigated the moderating effect of participants’ ethnic identity on the MS effect as an exploratory question. According to TMT, individuals attempt to allay their existential anxiety by espousing a specific cultural worldview. A previous study demonstrated that when faced with MS, individuals sought to defend the social groups that they identified with at that moment [15]. Because ethnic identity is interpretable as the degree to which individuals adhere to the cultural worldviews of their ethnic group, it is possible that the ethnic bias of individuals with strong identities would be reinforced to a greater extent in response to MS compared with those of individuals with weak ethnic identities.

To test our hypothesis, we conducted a pre-registered experiment with a large sample. Although past TMT studies using the IAT demonstrated a medium to large effect of MS [12, 13], recent criticism of the TMT has argued that past studies overestimated the effect size of MS [5]. Thus, we recruited a large number of participants using crowdsourcing to increase our ability to detect even a small effect.

## Main text

### Methods

We recruited 895 Japanese adults with no history of mental disorders (656 men and 230 women, nine unknown; *M*_age_ = 48.55) to participate in the experiment. Participants were recruited from a crowdsourcing platform in exchange for a reward of 40 Japanese yen (approximately US$0.3). To determine the sample size, we conducted a power analysis for independent samples t-tests using G*power 3.1 [16]. The analysis indicated that 788 participants were needed to detect a small effect (Cohen’s *d* = 0.2) with a power of 0.8 and α = 0.05. Thus, we recruited 900 participants, assuming that some would be excluded from the analysis.^1^

We designed the experiment with reference to past TMT studies [12, 13, 17]. The experimental program was created using jsPsych [18]. Participants were randomly assigned to either the MS or control condition. They were first informed that the purpose of the experiment was to investigate the relationships between an individual’s sporting experience and their values and identity.

After accessing the study webpage, participants answered four questions regarding their ethnic identity (e.g., “I am proud to be Japanese”) on a 7-point scale (1 = *not at all applicable* to 7 = *very applicable*) [19]. Then, participants assigned to the MS condition answered 20 items about their attitudes toward death (e.g., “I feel scared when I think about my existence ceasing.”) [20]. Participants assigned to the control condition answered 20 items about fear of dental treatment (e.g., “I have postponed visiting a dental clinic because of fear of dental treatment.”) [21]. Following the MS manipulation, participants completed the Japanese version of the Positive and Negative Affect Schedule scales [22] and answered filler questions. These items were used to create delays after the MS manipulation [23]. Finally, participants performed the IAT and were debriefed.

**Fig. 1:**
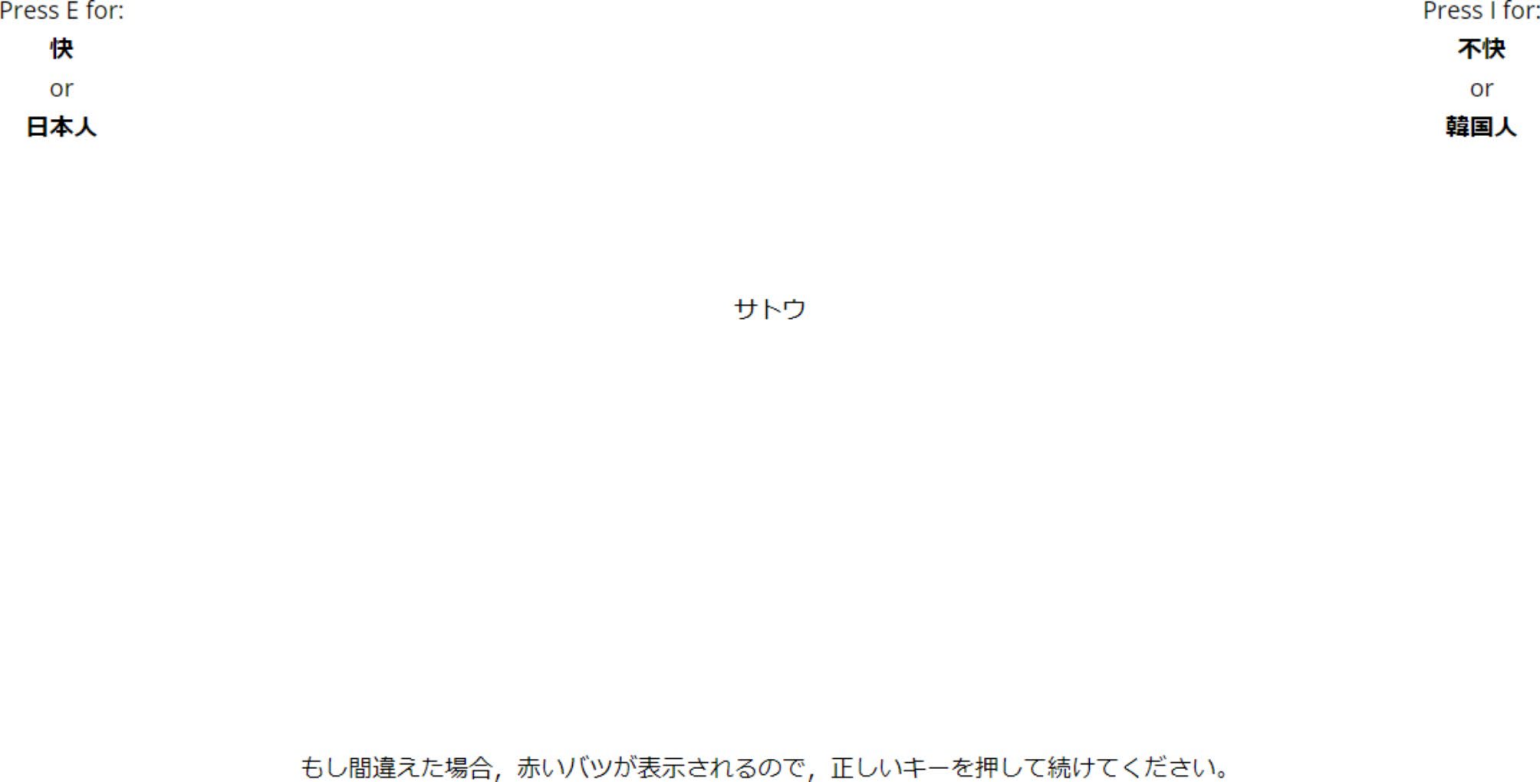
The IAT task

In this experiment, the IAT using Japanese and Korean surnames was performed to measure implicit ethnic bias among Japanese respondents (Fig. 1) [11]. In the IAT, participants sorted various words displayed on a screen into four categories (pleasant, unpleasant, Japanese, and Korean) using the “E” and “I” keys on the keyboard. The IAT comprised seven blocks. Blocks 4 and 7 each included 40 trials, whereas the others included 20 trials. Participants performed the first combined task in blocks 3 and 4, and performed the second task in blocks 6 and 7. There were two types of combined tasks: compatible and incompatible tasks. For the compatible task, participants classified compatible pairs of categories (pleasant and Japanese; unpleasant and Korean) by pressing the same key, whereas they were asked to form incompatible pairs of categories (unpleasant and Japanese; pleasant and Korean) by pressing the same key for the incompatible task. The order of performance of each task was counterbalanced.

The main dependent variable was the IAT effect. The IAT effect was defined as the mean response time for the incompatible task minus that for the compatible task [11]. We interpreted the IAT effect as the degree of participants’ implicit ethnic bias. The details of the IAT procedure are provided in the Appendix (Supplementary file 1).

The experiment was conducted in accordance with the code of ethics and conduct of the Japanese Psychological Association [24], after being pre-registered in the Open Science Framework [25].

## Results

We used R version 4.1.2 [26] for handling and analyzing the data.

**Fig. 2:**
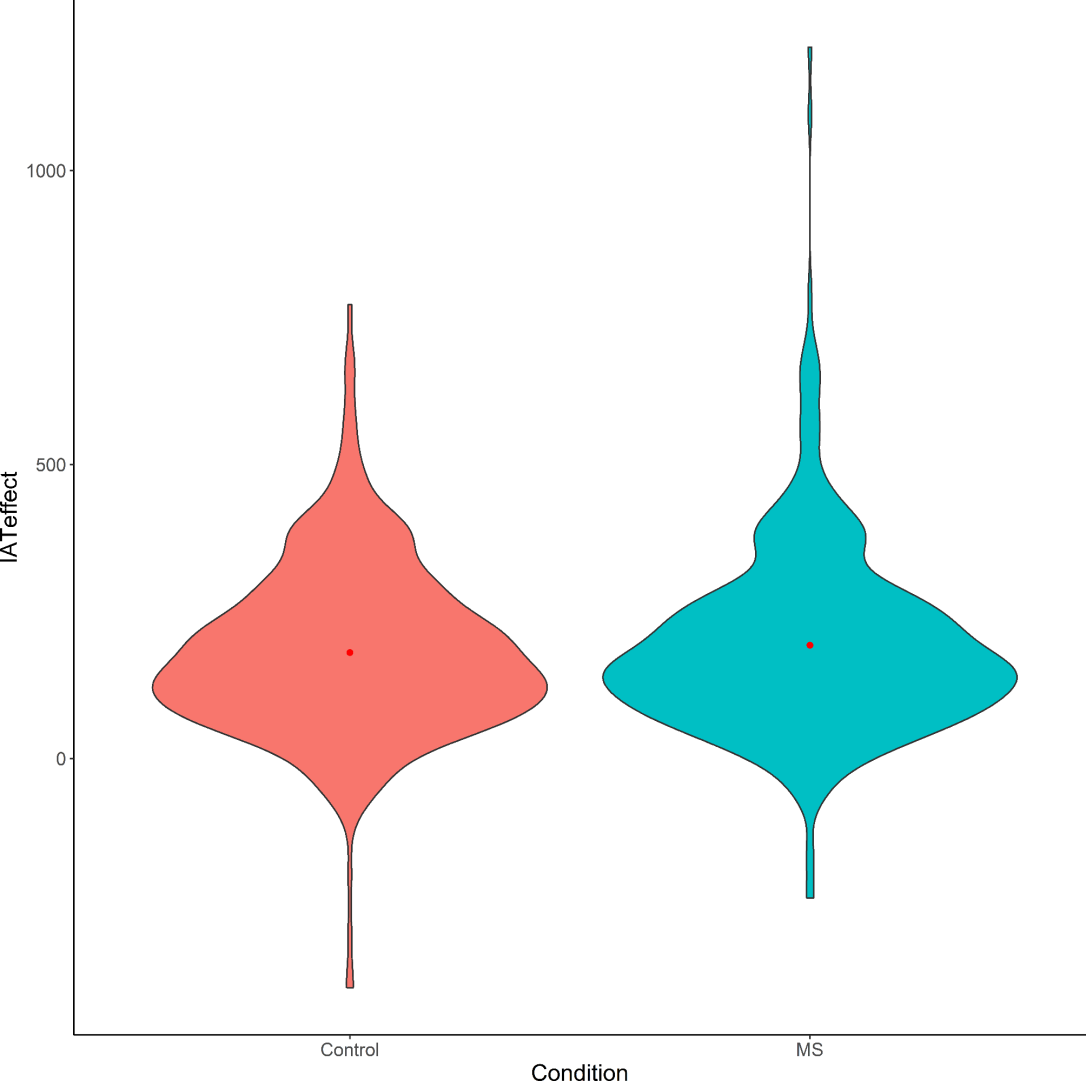
Distribution of the IAT effect (ms) in each condition Legends: Red dots indicate means

The response data from (1) 13 participants who responded to over 10% of the IAT trials in less than 300 ms [27], (2) 10 participants who left the IAT for more than 3 min, (3) two participants who reported that they participated in the same experiment in the past were excluded from the analysis. These criteria were all pre-registered. Responses to the four questions on ethnic identity were summed and treated as a single variable. We used IAT response times in blocks 4 and 7, excluding the first two trials and errors. We replaced values below 300 ms and above 3,000 ms with 300 ms and 3,000 ms, respectively [11]. Table 1; Fig. 2 show the means and SDs of response times in the IAT and the distribution of the IAT effect in each condition, respectively.

**Table 1:** Mean (SD) response times (ms) in the IAT

	Condition	
	Control	MS
Compatible task	756.96 (165.71)	744.24 (185.67)
Incompatible task	937.32 (215.55)	936.92 (252.37)

Before the analysis, we conducted a log transformation of response times [11]. To confirm that the IAT successfully measured participants’ implicit ethnic bias, a two-tailed paired t-test was performed on response times obtained in the compatible and incompatible tasks in the IAT. The results showed that the mean response time for the incompatible task was significantly longer than that for the compatible task in both conditions (MS: *t* (447) = 31.65, *p* < .001, *d* = 1.50, 95% CI: [1.36, 1.63]; Control: *t* (421) = 30.14, *p* < .001, *d* = 1.47, 95% CI: [1.33, 1.60]).

We calculated the IAT effect by subtracting the mean response time for the compatible task from that for the incompatible task. Then, to test H1, we conducted a two-tailed Welch’s t-test on the means of the IAT effect in each condition. The results revealed that there was no significant difference between them (*t* (867.5) = 1.19, *p* = .24, *d* = 0.08, 95% CI: [− 0.05, 0.21]). In addition, we conducted a multiple regression analysis with the IAT effect as the dependent variable; MS (dummy variable: MS condition = 1, control condition = 0), ethnic identity, and their interaction were the predictors. Predictors were centered to avoid multicollinearity. The results showed a significant effect of ethnic identity (*β* = 0.09, *t* (866) = 2.52, *p* = .01) and non-significant effects of MS (*β* = 0.04, *t* (866) = 1.11, *p* = .27) and their interaction (*β* = −0.02, *t* (866) = − 0.71, *p* = .48).

## Discussion

The analysis revealed that participants responded significantly faster when performing the compatible task than when performing the incompatible task in the IAT, indicating that their implicit ethnic bias was measured successfully. However, there was no significant difference between the means of the IAT effect in the MS condition and the control condition. Therefore, H1 was not supported. Additionally, we observed no moderating effect of participants’ ethnic identity on the IAT effect. These findings support the claim that East Asians do not exhibit worldview defense [6, 7], in accord with recent research questioning the validity of TMT [4, 5].

A previous study pointed out that most TMT studies could be underpowered, and that it is necessary to verify the predictions of TMT with an adequate sample size to detect a small effect [5]. In the current study, we examined the predictions of TMT with a large sample (N = 870) that was sufficient for detecting a small effect.^2^ The findings of this study have important implications for future investigations of TMT.

## Limitations

We recruited participants using a crowdsourcing platform in exchange for a small sum of money. It is possible that this procedure may have affected the quality of data as participants could have tried to complete the experiment as quickly as possible. However, the IAT response data indicated that there were only 13 participants (1.5%) who responded to over 10% of the IAT stimuli in less than 300 ms (i.e., those who appeared not to follow the instructions). Given that a previous study reported that 23–37% of participants recruited through Amazon Mechanical Turk performed similarly [28], the proportion of such respondents in our study appears to be relatively low. Thus, most participants seemed to have performed the experiment conscientiously.

Additionally, we cannot conclude that the absence of the MS effect in this study was caused by the limitations of the assumptions of TMT per se, or its applicability in East Asia, or other reasons from this experiment alone. Besides, previous studies pointed out that various factors such as age [29], attachment style [30], and cultural orientation [31] moderate the MS effect. Differences in such variables among participants might have yielded differing results in TMT studies. Future research should take this possibility into account and verify the validity of the effect in Eastern societies.

## Electronic supplementary material

Below is the link to the electronic supplementary material.

Supplementary Material 1

## Data Availability

The dataset supporting our findings is available in the OSF repository, https://osf.io/pds8t/files/osfstorage.

## Abbreviations

TMT: Terror management theory
MS: Mortality salience
IAT: Implicit Association Test

## Footnotes

1 Although we recruited 900 participants using a crowdsourcing platform, we obtained data from only 895 participants. This may have been caused by cheating (i.e., receiving the reward without participating) by five participants. Because the number of valid data sets was above the pre-registered target number (N = 788), we stopped data collection.

2 Despite recruiting a large enough sample to test the main effect of the MS, the test for the moderating effect of ethnic identity may have still been underpowered. We conducted a power analysis to determine the size of the detectable interaction effect in our multiple regression analysis using the InteractionPoweR package of R [32]. The result indicated that the analysis could detect an interaction effect of *f*^2^ = 0.009 with power of 0.8. Considering that a previous review reported that the median effect size of the interaction in past studies was *f*^2^ = 0.002 [33], it is highly likely that our analysis for the moderating effect was underpowered. Future research should verify the moderating effect of ethnic identity with an even larger sample.
