# Supplementary material for: No significant effect of mortality salience on unconscious ethnic bias among the Japanese
Source: BMC Res Notes. 2023 May 26;16:91. doi: 10.1186/s13104-023-06360-9 (PMC10214735; doi:10.1186/s13104-023-06360-9)
Supplement: Supplementary file 1 — Supplementary Material 1 [file 13104_2023_6360_MOESM1_ESM.docx]

**Appendix**

**The IAT**

**Procedure**

For the IAT, participants were asked to sort various words displayed on a screen into four categories (pleasant words, unpleasant words, Japanese surnames, and Korean surnames) using the “E” and “I” keys on the keyboard. If participants pressed the wrong key, a red “X” appeared on the screen, and they were instructed to press the correct one. The IAT comprised seven blocks. Blocks 4 and 7 each included 40 trials, whereas the others included 20 trials. For block 1, participants were asked to sort pleasant and unpleasant words. For block 2, they classified Japanese and Korean surnames. For blocks 3 and 4, they performed the first combined task. For block 5, participants sorted Japanese and Korean surnames in the reverse order to that created for block 2. For blocks 6 and 7, they performed the second combined task. We used the response data for blocks 4 and 7 in the analysis. There were two types of combined tasks: compatible and incompatible tasks. For the compatible task, participants were instructed to classify compatible pairs of categories (pleasant words and Japanese surnames; unpleasant words and Korean surnames) by pressing the same key, whereas they were asked to form incompatible pairs of categories (unpleasant words and Japanese surnames; pleasant words and Korean surnames) by pressing the same key for the incompatible task. The order of performance of each task was counterbalanced. Four hundred and thirty-three participants performed the compatible task first and the remaining participants performed the incompatible task first. The interval between trials was 200 ms. We predicted that the response time for the compatible task would be shorter than that for the incompatible task and deemed that the gap in response times between the two tasks (i.e., the IAT effect) would represent the degree of a participant’s implicit ethnic bias [1].

**Stimuli**

These items were partially adopted from Greenwald et al. [1].

- 快 (Pleasant): “楽しい,” “優しい,” “名誉,” “幸せ,” “友達” (“enjoyable,” “gentle,” “honor,” “happy,” “friend”)
- 不快 (Unpleasant): “醜い,” “悪い,” “憎しみ,” “病気,” “苦痛” (“ugly,” “evil,” “hatred,” “sickness,” “agony”)
- 日本人 (Japanese surnames): “タナカ,” “サトウ,” “ヤマダ,” “ニシ,” “オノ”(“Tanaka,” “Sato,” “Yamada,” “Nishi,” “Ono”)
- 韓国人 (Korean surnames): “ウォン,” “ヒョン,” “ユン,” “パク,” “チェ”(“Hwang,” “Hyun,” “Yoon,” “Paik,” “Choi”)

**References**

1. Greenwald AG, McGhee DE, Schwartz JL. Measuring individual differences in implicit cognition: the implicit association test. J Pers Soc Psychol. 1998. [https://doi.org/10.1037/0022-3514.74.6.1464](https://psycnet.apa.org/doi/10.1037/0022-3514.74.6.1464).
